# Single-cell RNA sequencing reveals recruitment of the M2-like CCL8^high^ macrophages in Lewis lung carcinoma-bearing mice following hypofractionated radiotherapy

**DOI:** 10.1186/s12967-024-05118-6

**Published:** 2024-03-25

**Authors:** Haonan Yang, Zheng Lei, Jiang He, Lu Zhang, Tangmin Lai, Liu Zhou, Nuohan Wang, Zheng Tang, Jiangdong Sui, Yongzhong Wu

**Affiliations:** 1 School of Medicine, Chongqing University, Chongqing, 400044 China; 2 Radiation Oncology Center, Chongqing University Cancer Hospital, No. 181 Hanyu Road, Shapingba District, Chongqing, 400030 China

**Keywords:** Hypofractionated radiotherapy, Macrophages, Chemokines, CCL8, CCL signaling pathway

## Abstract

**Background:**

Tumor-associated macrophages (TAMs) play a pivotal role in reshaping the tumor microenvironment following radiotherapy. The mechanisms underlying this reprogramming process remain to be elucidated.

**Methods:**

Subcutaneous Lewis lung carcinoma (LLC) murine model was treated with hypofrationated radiotherapy (8 Gy × 3F). Single-cell RNA sequencing was utilized to identify subclusters and functions of TAMs. Multiplex assay and enzyme-linked immunosorbent assay (ELISA) were employed to measure serum chemokine levels. Bindarit was used to inhibit CCL8, CCL7, and CCL2. The infiltration of TAMs after combination treatment with hypofractionated radiotherapy and Bindarit was quantified with flow cytometry, while the influx of CD206 and CCL8 was assessed by immunostaining.

**Results:**

Transcriptome analysis identified a distinct subset of M2-like macrophages characterized by elevated Ccl8 expression level following hypofractionated radiotherapy in LLC-bearing mice. Remarkbly, hypofractionated radiotherapy not only promoted CCL8^high^ macrophages infiltration but also reprogrammed them by upregulating immunosuppressive genes, thereby fostering an immunosuppressive tumor microenvironment. Additioinally, hypofractionated radiotherapy enhanced the CCL signaling pathway, augmenting the pro-tumorigenic functions of CCL8^high^ macrophages and boosting TAMs recruitment. The adjunctive treatment combining hypofractionated radiotherapy with Bindarit effectively reduced M2 macrophages infiltration and prolonged the duration of local tumor control.

**Conclusions:**

Hypofractionated radiotherapy enhances the infiltration of CCL8^high^ macrophages and amplifies their roles in macrophage recruitment through the CCL signaling pathway, leading to an immunosuppressive tumor microenvironment. These findings highlight the potential of targeting TAMs and introduces a novel combination to improve the efficacy of hypofractionated radiotherapy.

**Supplementary Information:**

The online version contains supplementary material available at 10.1186/s12967-024-05118-6.

## Background

Radiation therapy is an important modality for cancer treatment. In the case of non-small cell lung cancer (NSCLC), radiotherapy is the only treatment applicable to all TNM stages [1]. It is estimated that over 60% of NSCLC patients require radiotherapy during their treatment [2]. Hypofractionated radiotherapy, especially for the Stereotactic body radiation therapy (SBRT), is a precision treatment technique that targets the tumor while minimizing exposure to surrounding normal tissue. This allows for high-dose fractionated radiation, shortening treatment duration and improving therapeutic outcomes. Specifically, for inoperable early-stage NSCLC, hypofractionated radiotherapy significantly improves clinical outcomes compared to conventional radiotherapy and has become the current standard treatment protocol [3, 4]. Beyond its direct tumoricidal effects, hypofractionated radiotherapy also modulates immune response through various mechanisms including the release of tumor-associated antigens (neoantigens) and damage-associated molecular patterns (DAMPs), enhancement of antigen presentation, releasing of cytokines and chemokines for inflammatory response regulation, and promotion of T cell activation and infiltration [5–8]. However, hypofractionated radiotherapy also contributes to the recruitment of immunosuppressive cells such as FOXP3^+^ regulatory T cells (Tregs), tumor-associated neutrophils (TANs), tumor-associated macrophages (TAMS), and myeloid-derived suppressor cells (MDSCs) via a series of cytokines and chemokines, thereby inducing an immunosuppressive tumor microenvironment [9, 10]. In a recent phase 2 trial, the combination of SBRT with a PD-1 inhibitor markedly improved event-free survival in patients with early-stage NSCLC [4]. Together, it is crucial to investigate the mechanisms underlying the immunomodulatory effects of hypofractionated radiotherapy and to identify new therapeutic targets to improve treatment outcomes.

Tumor-associated macrophages are traditionally categorized into two phenotypes: M1 and M2. M1 macrophages are considered pro-inflammatory and play a role in inhibiting tumor growth, whereas M2 macrophages exhibit anti-inflammatory functions and are known to promote tumor progression [11, 12]. M2 TAMs form an important component of tumor immunosuppressive microenvironment [13, 14]. In the era of single-cell RNA sequencing, TAMs in NSCLC have been extensively studied. Wang et al. [15] reported that TAMs are more prevalent in the progression from normal lung tissue to stage IA LUAD. In end-stage LUAD, both primary tumors and brain metastases exhibit a significant enrichment of TAMs Furthermore, there is a gradual replacement of normal resident myeloid cells by monocyte-derived TAMs, creating an immunosuppressive microenvironment [16]. Corroborating these findings, tissue-resident macrophages have been implicated in promoting epithelial-mesenchymal transition and invasiveness in tumor cells, as well as in recruiting regulatory T cells during the early stages of lung cancer. These resident macrophages are not only supplanted by monocyte-derived macrophages but are also repositioned to the periphery of the TME as the tumor progresses [17]. Within the TAM subclusters, M2-like macrophages, with diminished antigen-presenting abilities, were predominantly found in early-stage LUAD tumors compared to matched adjacent and distal normal lung tissues [18]. In the case of lung squamous carcinoma (LUSC), macrophages expressing high levels of SPP1 constituted the main subtype of TAMs, a macrophage subcluster previously identified as angiogenesis promoters in various cancer types [19, 20]. Additionally, radiotherapy has been shown to promote the infiltration of TAMs into tumors which can attenuate the therapeutic efficacy of radiotherapy [5]. As a result, understanding the dynamics of TAMs in the context of radiotherapy and targeting TAMs to overcome this immunosuppressive effect is vital for enhancing treatment efficacy.

Chemokines are a group of cytokines that induce cell migration and participate in regulating inflammatory responses by binding to corresponding receptors. Based on the N-terminal cysteine residue sequence, chemokines are classified into four subtypes: CC, CXC, C, and CX3C, among which CC chemokines are further divided into 27 types. In the tumor microenvironment, CC chemokines mediate the infiltration and differentiation of immunosuppressive cells such as TAM, MDSC, TAN, Treg, and contribute to tumor immune evasion [21, 22]. Bioinformatics analysis of transcriptional profiles from LUAD patients in TCGA and GEO cohorts revealed that a predominant subset of these patients displayed an ‘inflamed’ immune phenotype. This phenotype is marked by the upregulation of various chemokine genes, such as CCL2, CCL5, CCL7, and CCL8. Moreover, this subtype with elevated expression of chemokine genes is associated with decreased survival rates [23]. Current research also finds that radiotherapy recruits immunosuppressive cells to reshape the tumor microenvironment, weakening anti-tumor immunity, with CC chemokines playing a significant role in this process [24]. Specifically, CCL2 (C–C motif ligand 2) is most clearly related to the post-radiotherapy immune microenvironment. It not only recruits immunosuppressive cells such as tumor-associated macrophages, myeloid-derived suppressor cells (MDSC) but also induces macrophage polarization and further secretion of cytokines [25, 26]. Unlike CCL2, the release of CCL5 by tumor cells not only promotes TAMs and MDSCs infiltration, participating in tumor metastasis [27],but also recruits CD8 + T cells to enhance adaptive immunity. This demonstrates its dual role in immune regulation [28]. CCL7 is reported to cause polarization of macrophage towards the M1 phenotypeand to induce radiation-induced lung injury [29]. Although it is established that CCL8 promotes tumor cell proliferation and migration, its role on the tumor microenvironment following radiotherapy are not yet fully understood [30, 31].

In the present study, we profiled early alterations of tumor microenvironment in a subcutaneous (s.c.) Lewis lung carcinoma (LLC) murine model following hypofractionated radiotherapy by performing single-cell RNA sequencing. We then identified a M2-like CCL8^high^ macrophage population which contributed to immune suppression. Moreover, hypofractionated radiotherapy promoted this specific macrophage population infiltration and reprogrammed TAMs through CCL signaling pathway. The CCL signals inhibitor, Bindarit, synergized with hypofractionated radiotherapy to extend local control in our s.c. LLC murine model.

## Methods

### Cell lines and drugs

Lewis lung carcinoma (LLC) cell line was purchased from the National Collection of Authenticated Cell Cultures, China. Cells were cultured at 37 °C humidified incubator with 5% CO_2_ in DMEM medium supplemented with 10% FBS and 100 units/ml penicillin and 0.1 mg/ml streptomycin. The cells were routinely tested to confirm the absence of Mycoplasma contamination and were cultured for a limited number of generations. The CCL8 inhibitor Bindarit (AF2838, MCE, Cat#: HY-B0498) was diluted in 10% DMSO, then 45% PEG300 (MCE, Cat#: HY-Y0873) and 45% Saline. Recombinant CCL8 protein was purchased from MCE (Cat. #: HY-P7771).

### Mice, in vivo studies, and treatments

Female C57BL/6N mice ages 6 to 8 weeks were purchased from Beijing Vital River Laboratory Animal Technology. All animal procedures followed the Guide for the Care and Use of Laboratory Animals. For the LLC xenograft tumor models, 1 × 10^6^ LLC cells in 100ul PBS were injected subcutaneously in the right flank of C57BL/6 mice. For radiotherapy, once tumors reached an average volume of 100 mm^3^, radiation was given on day 7–9 post-tumor cell injection with total dose 24 Gy in 3 fractions (8 Gy each fraction). For CC chemokines inhibitor, Bindarit was administered intraperitoneally 100 mg/kg daily from day 5 post-tumor injection and was continued for 7 days. Tumors were measured with a caliper and mice were euthanized when tumor volumes reached 1500 mm^3^. Weight was monitored three times a week.

### Cell preparation and single-cell RNA sequencing

Tumors were dissociated using Multi Tissue Dissociation Kit 2 (Miltenyi Biotec, Cat#: 130–110-203) according to manufacturer’s protocol. Debris and dead cells were removed (Miltenyi Cat#: 130–109-398/130–090-101). Fresh cells were resuspended at 1 × 10^6^ cells per ml in 1 × PBS and 0.04% bovine serum albumin. Single-cell RNA-Seq libraries were prepared using SeekOne MM Single Cell 3′ library preparation kit following the manufacturer’s instructions. Sequencing was performed on the Illumina NovaSeq 6000 with PE150 read length. The SeekOne Tools pipeline was used to process the cleaned reads and generated the transcript expression matrices. The schematic workflow for single-cell RNA sequencing was generated by using the Biorender software (www.biorender.com).

### Clustering scRNA-seq data and cell type annotation

All additional analyses were performed using R 4.3.0. Unsupervised clustering was performed by Seurat package [32] (version 4.3.0) and cells were integrated using the Harmony [33]. After that highly variable genes (HVGs) were selected for principle components analysis (PCA), and the top 30 significant principal components (PCs) were selected for Uniform Manifold Approximation and Projection (UMAP) and t-distributed stochastic neighbor embedding (tSNE) dimension reduction, and visualization of gene expression. Next, differentially expressed genes (DEGs) of each cell subcluster were identified by running the “FindAllMarkers” function of Seurat package. Final, cell types were annotated according to the expression level of the known canonical marker genes of certain cell types. Transcription factor activity analysis based on DEGs was conducted by using the DoRothEA regulatory network analysis [34].

### Enrichment analysis

DEGs which |logFC|> 0.5 and adjusted P value < 0.05 were selected for further analysis. The clusterProfiler package [35] was used to process GO and KEGG enrichment between macrophage subclusters with or without treatment. Gene set variation analysis of hallmark gene sets (MSigDB) for tumor subclusters was conducted using the GSVA [36].

### Pseudotime analysis

To analyze the gene characteristics of myeloid cells differentiation, pseudotime analysis was performed by monocle2 package [37]. The dpFeature method provided by the differentialGeneTest function was applied to find DEGs for pseudotime analysis. The function called plot_genes_in_pseudotime and plot_pseudotime_heatmap were applied for visualization.

### Cellchat analysis

To explore cell–cell communication between tumor cells and myeloid cells, the Cellchat package [38] was first used to calculate the communication probability of control and IR group separately. Then comparison analysis was applied with the mergeCellChat function to identify the upregulated and downregulated signals in IR group.

### TCGA data analysis

The TCGAbiolinks package was used to download the expression data of TCGA. For the gene signature, we used the three marker genes Ccl8, C1qb, and Fth1. The survival analysis was conducted in accordance with the methods reported by Cheng et al. in their study [20].

### Flow cytometry analysis

Tumors were collected 3 days after last radiation fraction then were dissociated in DMEM supplemented with 1 mg/ml Collagenase IV (Solarbio) and 1 μl/ml DNase I (Solarbio) at 37 °C for one hour. Single-cell suspensions were collected after filtering digested tissues with 40-um filter and washed with PBS supplemented with 2% FBS. Subsequently, cell surface and intracellular markers were stained with the following fluorochrome-conjugated antibodies: anti-CD45 BV421 (Cat#: 103,134), anti-CD11b PE (Cat#: 101,207), anti-F4/80 AF700 (Cat#: 123,129), anti-CD86 FITC (Cat#: 105,109), anti-CD206 APC (Cat#: 141,708), anti-CD4 BV510 (Cat#: 100,553), anti-CD8a AF700 (Cat#: 100,729) from Biolegend. For surface staining, all samples were stained with antibodies at 4 °C for 30 min; for intracellular staining, cells were fixed and permeabilized with BD Cytofix/Cytoperm (Cat#: 554,714) then stained according to the manufacturer’s instructions. Analysis of stained cells was performed using a CytoFlex cytometer (Beckman Coulter) and CytExpert software.

### Cytokine and chemokine assays

Blood of mice bearing LLC tumors were collected 3 days after last radiation fraction. Serum was diluted five times in PBS. Cytokines were quantified according to the manufacturer’s protocol (Luminex Mouse Discovery Assay, R&D Systems). CSF1, CCL2, CCL7, and CCL8 were further quantified by Elisa analysis according to manufacturer’s protocol (MEIMIAN, Cat#: MM-1025M1, MM-0082M1, MM-0084M1, and MM-0083M1).

### real-time qPCR

Cells were kept frozen (− 80 ℃) until mRNA extraction. The RNeasy kit and the cDNA Synthesis Kit (Takara, Cat#: 9767, RR047A) was used to extract total RNA and prepare cDNA following the manufacturer’s instructions. Real-Time qPCR was performed with BioRad PCR system. Primers for mouse Csf1 were 5′- TAGAAAGGATTCTATGCTGGG-3′ and reverse 5′- CTCTTTGGTTGAGAGTCTAAG-3′ (PrimerBank). They were purchased from Sangon Biotech (Shanghai, China).

### Immunostaining

Tissues were fixed with 4% paraformaldehyde for 72 h at 4 °C before being embedded. Immunostaining was performed using the multiplex immunohistochemistry kit from the AiFang biological (China, catalog Cat#: AFIHC035). The following primary antibodies were used: rabbit anti-F4/80 (AiFang biological, Cat#: SAF002), rabbit anti-CD206 (AiFang biological, Cat#: AF07082), rat anti-CCL8 (R&D, Cat#: MAB790). Nuclear staining was performed with DAPI. Images from the stained slides were scanned using the Digital Pathology Slide Scanner (KFBIO, China).

### Cell proliferation assay and colony formation assay

For proliferation assay and colony formation assay, the LLC cell line was treated with recombinant CCL8 (MedChemExpress, Cat#: HY-P7771) at 0, 5 ng/ml, 20 ng/ml. Then proliferation was assessed with a Cell Counting Kit-8 (Sigma-Aldrich, Cat#: 96,992) following manufacturer’s protocol. For colony formation assay, LLC cells were seeded into 6-well plates. The cell density for each cell was 500. After treating with recombinant CCL8 for 48 h, the culture medium was replaced by fresh DMEM. Cells were further incubated for another 7 days.

### Statistical analysis

Statistical analyses were conducted by R version 4.3.0 and GranphPad Prism 8.0. Multiple comparisons such as tumor growth curves were analyzed by two-way ANOVA. Violin plots comparing the gene expression level between two groups were analyzed by unpaired two-sided Wilcoxon test. Bar charts with mean value were analyzed by student’s t tests. P values less than 0.05 were considered statistically significant.

## Results

### Single-cell transcriptomic landscape of subcutaneous LLC tumors at early-stage following hypofractionated radiotherapy

**Fig. 1:**
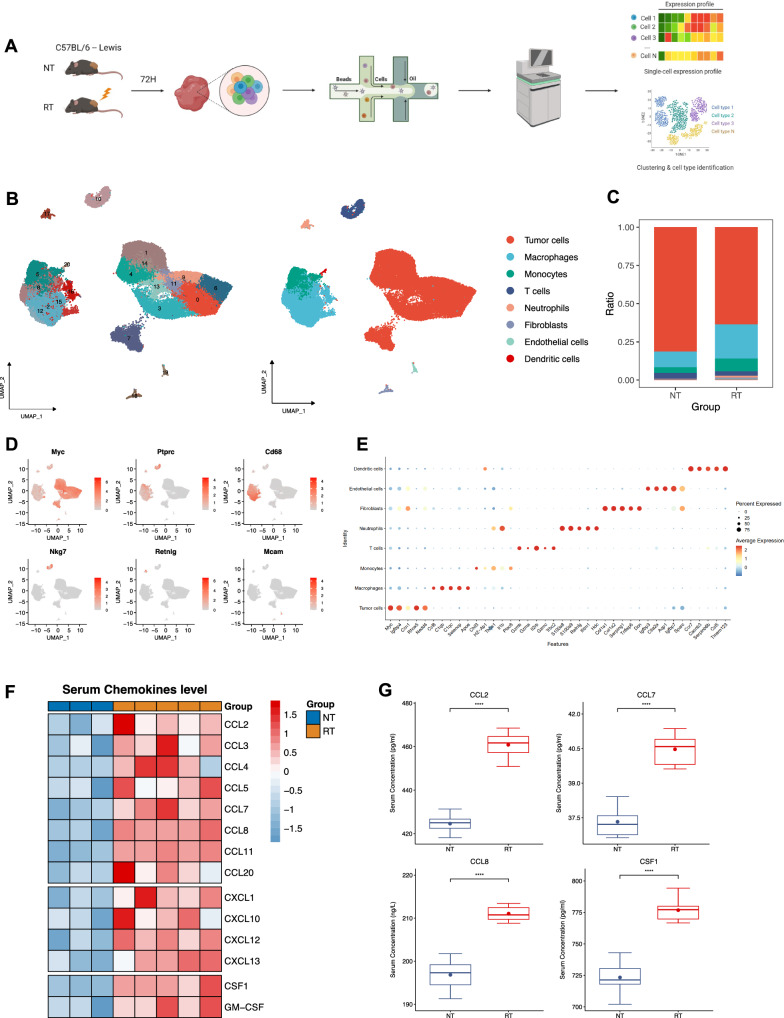
Single-cell transcriptomic landscape of subcutaneous LLC tumors at early-stage following hypofractionated radiotherapy. **A** Overview of the experimental design for single-cell RNA sequencing. **B** Uniform Manifold Approximation (UMAP) plot showing the unsupervised clusters of 54883 single cells and annotated cell types. **C** The proportion of each cell types in LLC tumors treated with or without radiation. For B and C, each color represents the same cell type. **D** Feature plot and **E** Dot plot showing marker genes. **F** Heatmap showing serum chemokines level in LLC murine models 72 h post-treatment. Each row in the heatmap has been scaled. **G** The concentrations of CCL2, CCL7, CCL8, and CSF1 in serum measured by ELISA (n = 5/group). Data are presented as mean ± SD with student unpaired t test. *P < 0.05; **P < 0.01; ***P < 0.001; ****P < 0.0001; ns, not significant

To investigate early response of the tumor microenvironment (TME) in lung cancer treated with hypofractionated radiotherapy, single-cell RNA sequencing (scRNA-seq) was performed to 4 subcutaneous LLC tumors at 72 h following radiation (Fig. 1A). Applying established quality control methods, 54,883 cells were obtained, including 44,919 cells from the hypofractionated radiotherapy group (3 individual samples) and 9964 cells from control mice (3 samples mixed in 1)(6). All cells were divided into 21 clusters by running the Seurat pipeline, then we identified 8 populations, including tumors cells, macrophages, monocytes, T cells, neutrophils, fibroblasts, endothelial cells, and dendritic cells by applying canonical gene signatures and SingleR package (Fig. 1B, D). Marker genes of each cell type were shown by a dot plot (Fig. 1E). Tumor cells were further confirmed by applying the inferCNV analysis. Comparing with macrophages and monocytes (reference cells), tumor cells showed obviously higher frequency of copy number variation (Additional file 1: Fig. S1C). As expected, the ratio of tumor cells decreased after hypofractionated radiotherapy and T cells infiltration was minimal in two groups. Intriguingly, macrophages were highly enriched in hypofractionated radiotherapy treated s.c. LLC tumors (22.41% for radiation treatment, 10.19% for radiation naive control) (Fig. 1C).

To further explore the early impact of hypofractionated radiotherapy on myeloid cells recruitment-related chemokines in lung cancer, multi-chemokines analysis was conducted to evaluate the release of serum chemokines of s.c. LLC bearing mice at 72 h after hypofractionated radiotherapy. We found that radiation (RT) at subcutaneous tumor site triggered the release of C–C and CXC chemokines at early-stage compared to radiation naive (NT) group (Fig. 1F). Consistently, a markedly increased CCL2, CCL7, and CCL8 serum levels after radiation were further determined using the enzyme-linked immunosorbent assay (ELISA) kits (Fig. 1G).

### The alteration of LLC cell states following hypofractionated radiotherapy

**Fig. 2:**
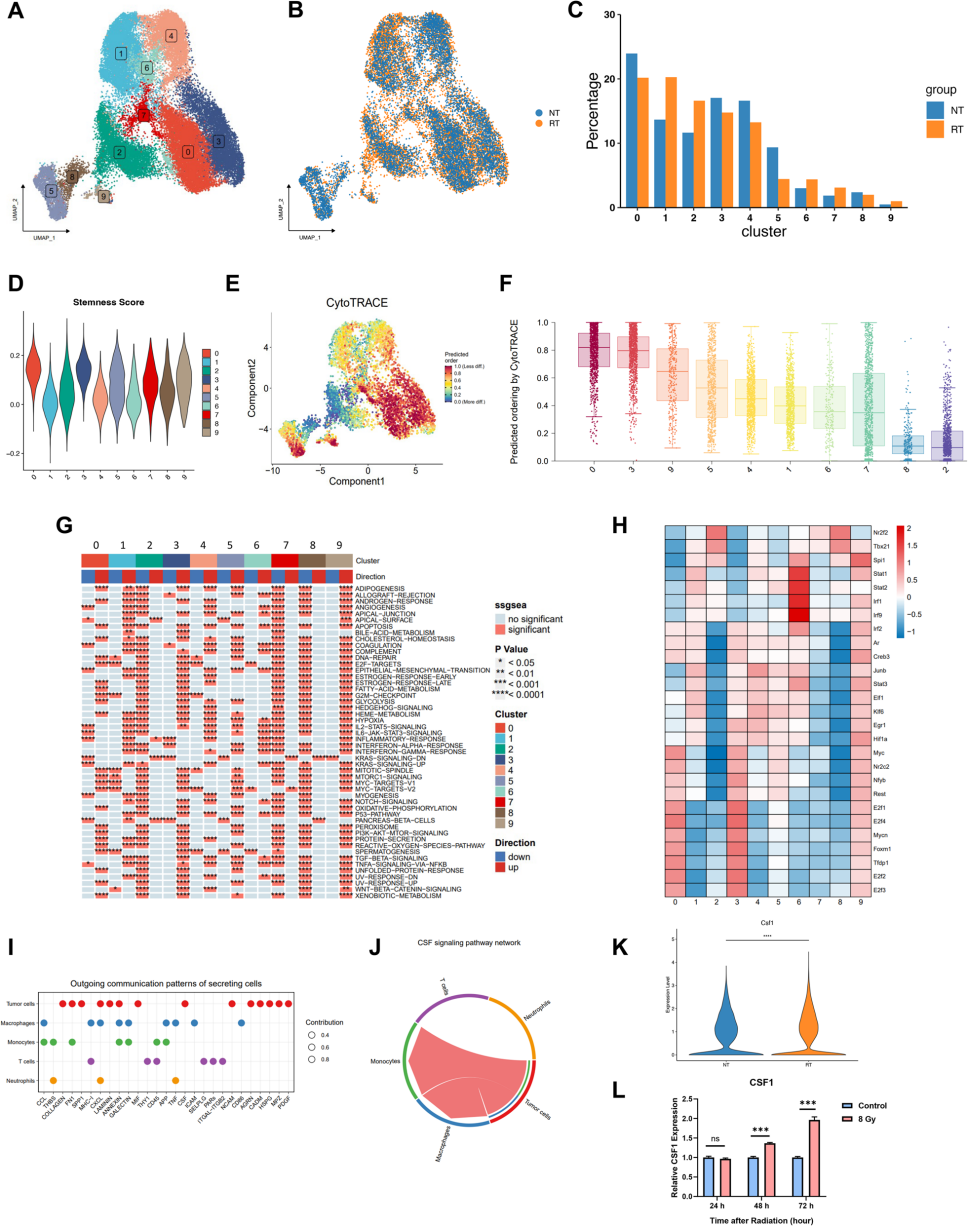
The alteration of LLC cell states following hypofractionated radiotherapy. **A** UMAP plot showing clusters of LLC cells. **B** UMAP plot showing the distribution of LLC cells in each group. **C** The proportion of each cluster in control (NT) and radiotherapy (RT) groups. **D** Violin plot showing stemness score for each cluster. **E** UMAP plot and **F** Bar plot of stemness in LLC cells predicted by CytoTRACE. **G** Heatmap of hallmark gene sets from the MSigDB enriched in different types of cell clusters. **H** Heatmap of transcription factors activity in each cell cluster. **I** Dot plot showing outgoing communication signaling pathways of different cell types. **J** Chord plot of communication network of CCL signaling pathway between tumor cells and other cell types. **K** Csf1 gene expression level between NT and RT groups. **L** The relative mRNA expression levels of Csf1 in LLC cells with or without exposure to 8 Gy of radiation in vitro. Data are representative of three independent experiments

Performing unsupervised dimensionality reduction and clustering to previously annotated tumor cells, we identified 10 tumor cell populations with distinct gene expression profiles (Fig. 2A). The distribution of tumor cell populations was identical in two groups after batch effect correction (Fig. 2B). However, cluster 0 and cluster 3 to 5 were less abundant in RT group, while cluster 1, 2, 6 and 7 were enriched after RT (Fig. 2C). We then evaluated the stemness of each cell population by calculating the expression score of a curated stemness gene signature [39]. Cluster 0 and 3 demonstrated the highest stemness score among all LLC cells (Fig. 2D). These findings were further corroborated using CytoTRACE, a computational algorithm that predict cellular differentiation state, with higher score indicating greater stemness [40]. Based on the differentiation score, cluster 0 and 3 exhibited highest level of stemness (Fig. 2E, F), whereas cluster 2 and 8 were identified as the most differentiation tumor cells. Gene set variation analysis (GSVA) revealed that cluster 0 were enriched for expression genes related with DNA repair, cell cycle (Hallmarks E2F-Targets, G2M checkpoint), MYC targets, and Oxidative phosphorylation, which implied higher intrinsic radiation sensitivity. Along with enrichment of DNA repair and cell cycle genes, cluster 3 exhibited higher expression level of epithelial-mesenchymal transition (EMT) related genes. Conversely, cluster 2 and 8 downregulated most hallmark gene sets compared to other clusters (Fig. 2G). Further gene expression profiling analysis confirmed the upregulation of cell cycle genes of cluster 0 and 3. Intriguingly, cluster 6, which exhibited moderate differentiation state, was found highest expression level of transcription factors STAT1/STAT2-IRF9/IRF1 activated by interferon gamma (IFN-γ) leading to upregulation of tumor PD-L1 expression following RT (Fig. 2H). Cell–cell communication analysis between tumor cells and immune cells revealed a specific CSF (colony stimulating factor) signaling pathway recruiting monocytes and macrophages since only tumor cells expressing the ligand gene Csf1 and barely expressing Csf1 receptor gene (Fig. 2I, J and Additional file 1: Fig. S1D). Moreover, radiation augmented expression of Csf1 in LLC cells and its release in mouse serum (Fig. 1G, 2 K). This was further demonstrated by reverse transcription quantitative PCR (RT-qPCR) (Fig. 2L). These results suggest that LLC maintains heterogeneity in murine s.c. tumor model and enables us to understand the unique transcriptomic characteristics of subpopulations of LLC cells following radiation. Notably, hypofractionated radiotherapy observably promotes CSF signaling pathway, which is supposed to mediate myeloid cells development [41].

### Hypofractionated radiotherapy stimulates a distinctive immune microenvironment at early-stage

**Fig. 3:**
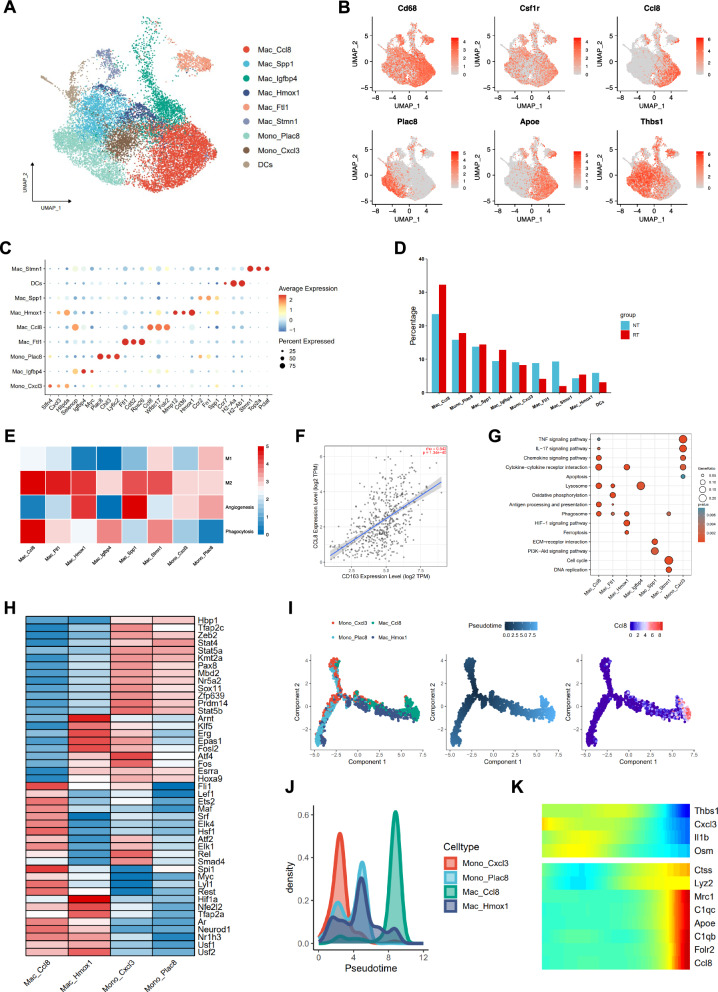
Identification of M2-like Ccl8^high^ Macrophages in the LLC-bearing murine model. **A** UMAP plot showing the annotation of macrophage populations in LLC tumors. **B** Feature plot and **C** Dotplot showing marker genes of macrophage populations. **D** Cell proportion of each cell type in NT and RT groups. **E** Heatmap displaying scores of M1, M2, angiogenesis, phagocytosis for each macrophage population. **F** Correlation analysis of CCL8 and CD163 expression level in TCGA-LUAD datasets (Spearman’s rho value = 0.542, p < 0.001). **G** The Gene Ontology (GO) enrichment analysis of each macrophage populations. **H** Heatmap displaying transcription factors activity of the Mac_Ccl8, Mac_Hmox1, Mono_Cxcl3, and Mono_Plac8 populations. **I** Development trajectory of macrophages populations predicted by Monocle2. **J** The cell density and **K** gene expression patterns along with the pseudotime,

To investigate the influence of hypofractionated radiotherapy on myeloid cells recruitment at early-stage in s.c. LLC tumors, unsupervised clustering and manual annotation identified 6 macrophages clusters (Mac_Ccl8, Mac_Spp1, Mac_Igfbp4, Mac_Hmox1, Mac_Ftl1, and Mac_Stmn1), 2 monocytes clusters (Mono_Plac8 and Mono_Cxcl3) and a cluster of DCs with distinct gene expression pattern (Fig. 3A, C). Marker gene feature plots further validated cell type annotation (Fig. 3B). Apoe, representing lipid-associated macrophage or TREM2 macrophage, was mainly expressed in Ccl8^high^ macrophage (Mac_Ccl8), while monocyte or monocyte-derived cells gene, such as Thbs1, was expressed in Mono_Plac8, Mono_Cxcl3 and Mac_Spp1. Notably, Mac_Ccl8 was the major population of tumor-infiltrating myeloid cells, and hypofractionated radiotherapy induced enrichment of Mac_Ccl8 compared to NT (32.28% in RT versus 23.48% in NT) (Fig. 3D). Using M1/M2 macrophages gene signatures [20], we found M2 was the dominant polarization state of myeloid cells in LLC-bearing mice. Moreover, Mac_Ccl8 not only exhibited highest M2 signatures score among all TAMs populations but also expressed higher phagocytosis genes relative to other populations (Fig. 3E). In addition, the significant correlation of Ccl8 and M2 macrophages was then demonstrated by applying TIMER 2.0 based on TCGA-LUAD expression profile (Fig. 3F). Consistently, the Gene Ontology (GO) enrichment analysis indicated upregulation of the chemokine signaling pathway, cytokine-cytokine receptor interaction, antigen processing and presentation, and phagosome gene sets of Mac_Ccl8 (Fig. 3G), indicating its multifarious functional features. When compared the transcriptomic profiles between Mac_Ccl8 and other myeloid cell types, we found that Mac_Ccl8 highly expressed a series of transcription factors, such as Spi1, Maf (c-Maf) and Fli1 (Fig. 3H). Spi1 and Maf are reported to regulate the transcriptional activity of CSF1R, and Maf enhances Ccl8 promoter activity at the transcription level [42–44]. Then, applying single cell trajectory analysis, we found that Mac_Ccl8 was potentially derived from monocytes. Pseudotime analysis revealed later evolution state of Mac_Ccl8 when monocytes were set as the progenitor (Fig. 3I, J). Along this trajectory, the expression level of Ccl8 gene rapidly upregulation at the end state, other genes, including Mrc1, Apoe, C1qb, C1qc, and Folr2 also exhibited same expression pattern*,* whereas expression level of Fn1, Chil3, and Thbs1 genes were higher in early state (Fig. 3K).

**Fig. 4:**
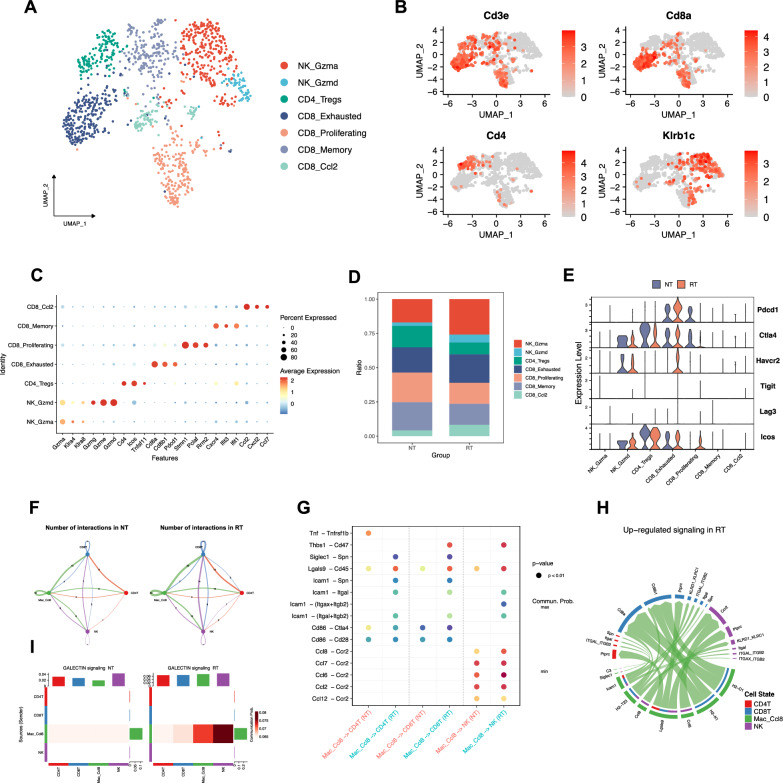
Hypofractionated radiotherapy promoted the crosstalk between the Mac_Ccl8 and lymphocytes. **A** UMAP plot showing the annotation of lymphocyte populations. **B** Feature plot and **C** Dot plot showing marker genes of lymphocyte populations. **D** The proportion of each lymphocyte population in two groups. **E** Violin plots displaying the expression level of immune checkpoint ligand genes in each lymphocyte populations of NT and RT groups. **F** Circle plots showing number of interactions in two groups inferred by CellChat. **G** The comparison of cellular communication probability from Mac_Ccl8 to T and NK cells in between two groups. **H** Chord plot displaying the upregulated signaling pathways in the RT group relative to the NT group. **I** Heatmap of the differential interaction strength of the GALECTIN signaling pathway between two groups

In addition, we identified 7 lymphocyte populations based on their transcriptomic files including 2 NK cell populations (NK_Gzma, NK_Gzmd), 4 populations of CD8 + T cell (CD8_Proliferating, CD8_Memory, CD8_Exhausted, and CD8_Ccl2), and a CD4_Treg populations (Fig. 4A, C). The proportion of NK_Gzma, NK_Gzmd and CD8_Exhausted populations was higher in the RT group, while the proportion of CD4_Tregs, CD8_proliferating, CD8_memory populations was higher in the NT group (Fig. 4D). Notably, hypofractionated radiotherapy upregulated immune check point genes PD-1, CTLA-4, TIM-3 (Mus musculus Havcr2) in exhausted T cells (Fig. 4E, Additional file 1: Fig. S3C). Cell–cell communication analysis revealed that Mac_Ccl8 inhibited CD4 and CD8 T cells activity via immune checkpoint ligand Lgals9 and Cd86 while recruiting NK cells by CCL signaling pathway (Fig. 4G). Furthermore, hypofractionated radiotherapy promoted the crosstalk between Mac_Ccl8 and other cell types (Fig. 4F, H). For instance, the galectin signaling pathway, including Lgals9 and its receptor Cd44, Cd45, and Havcr2 was significantly upregulated following hypofractionated radiotherapy (Fig. 4I).

### Hypofractionated radiotherapy reprograms CCL8^high^ macrophages through the CCL signaling pathway

**Fig. 5:**
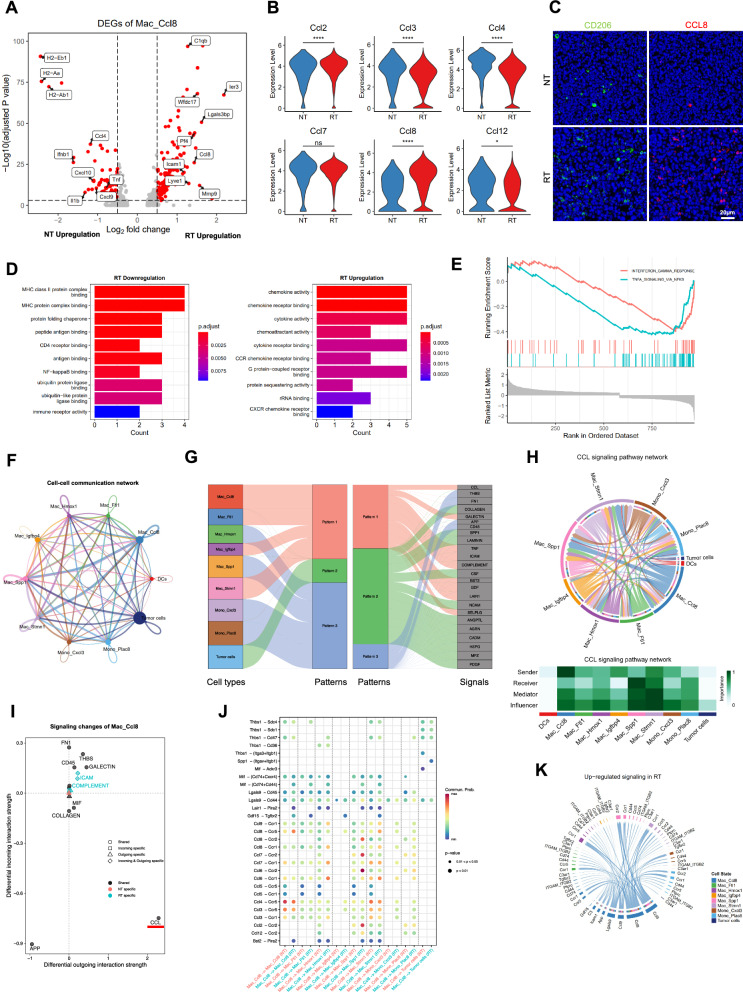
Hypofractionated radiotherapy reprograms CCL8^high^ macrophages through the CCL signaling pathway. **A** Volcano plot showing differentially expressed genes of the Mac_Ccl8 between the NT and RT groups. Adjusted p value < 0.05, two-sided Wilcoxon test. **B** Violin plots comparing the expression of Ccl2, Ccl3, Ccl4, Ccl7, Ccl8, and Ccl12 in the NT and RT groups. Unpaired two-sided Wilcoxon test. **C** Representative examples of multiplex immunofluorescent labeling CD206 and CCL8. Green, CD206; Red, CCL8; Blue, DAPI. **D** Bar plots showing the GO enrichment analysis of upregulation and downregulation genes in the RT group. **E** Differences in IFN-Gamma and TNF pathways activity between two groups inferred by GSEA. **F** Cell–cell communication network between myeloid populations and LLC cells. **G** River plot displaying communication patterns of different cell types. **H** Chord plot (top) and heatmap (bottom) showing communication network of CCL signaling pathway in different cell types. **I** Weighted network analysis of differential interaction strength of signals in the Mac_Ccl8 population between two groups. **J** The comparison of cellular communication probability from Mac_Ccl8 to other myeloid populations between two groups. **K** Chord plot displaying the upregulated signaling pathways in the RT group relative to the NT group. *P < 0.05; **P < 0.01; ***P < 0.001; ****P < 0.0001; ns, not significant

We sought to investigate whether hypofractionated radiotherapy altered Mac_Ccl8 states. Differentially expressed genes (DEGs) analysis showed that radiation upregulated immune suppressive genes C1qb, Mmp9, and Lgals3bp; pro-phagocytosis gene Icam1; and pro-angiogenesis gene Lyve1, whereas radiation downregulated MHC-II genes H2-Eb1, H2-Aa, and H2-Ab1; pro-inflammatory genes Tnf, Ifnb1, and Il1b (Fig. 5A). For chemokines, hypofractionated radiotherapy triggered expression of Ccl8 and Ccl, but downregulated the levels of Ccl3, Ccl4, and Ccl12 (Fig. 5B). Additionally, the immunostaining demonstrated an upregulation of CD206 and CCL8 in LLC tumors after radiation (Fig. 5C). The GO enrichment analysis of DEGs revealed the enhanced chemokine-activity and chemokine receptor binding, but reduction in antigen presentation of Mac_Ccl8 cluster after treating with hypofractionated radiotherapy (Fig. 5D). Gene set enrichment analysis (GSEA) validated downregulation of IFN-γ and TNF signaling in RT Mac_Ccl8, indicating that radiation contribute to its anti-inflammatory function and M2-like polarization (Fig. 5E).

Next, cell–cell communication network detected 26 signaling pathways between tumor cells and myeloid cells, and that Mac_Ccl8 was the major source communicating each other in macrophage populations (Fig. 5F). We then recognized 3 patterns of those signaling pathways. As shown in Fig. 5G, Mac_Ccl8 exhibited unique outgoing cellular communication pattern, and the cellular communication pattern 1 featured chemokine signaling pathway (CCL, specifically referring to C–C chemokine in this context). Mac_Ccl8 was identified as the strongest signaling sender whereas Mac_Spp1 was the most significant signaling receiver. Tumor cells were barely involved in cellular communication mediated by CCL signaling pathway (Fig. 5H). Furthermore, radiation elevated number and strength of interactions between cells (Additional file 1: Fig. S2G, H). Differential interaction strength analysis demonstrated, as expected, radiation enhanced the communication strength through CCL signaling pathway (Fig. 5I). As the major source of CCL signal, radiation augmented communication probability from Mac_Ccl8 to monocyte and macrophage populations including Mac_Ccl8 itself through interaction between CCL8 and CCR receptors (Fig. 5J, K). Moreover, we found that recombinant CCL8 protein at concentrations of 5 ng/ml and 10 ng/ml did not inhibit the viability of LLC cells in vitro. Similarly, adding recombinant CCL8 protein did not affect the colony formation of LLC cells. Thus, Mac_Ccl8 did not promote LLC growth directly (Additional file 1: Fig. S4A, B). Overall, hypofractionated radiotherapy reprogramed CCL8^high^ macrophages toward an immune suppressive state via strengthened CCL signaling-dependent cellular communication between each subset of myeloid cells (see Additional file 2).

### Hypofractionated radiotherapy promotes M2-like Ccl8^high^ macrophages infiltration and leads to poor prognosis

**Fig. 6:**
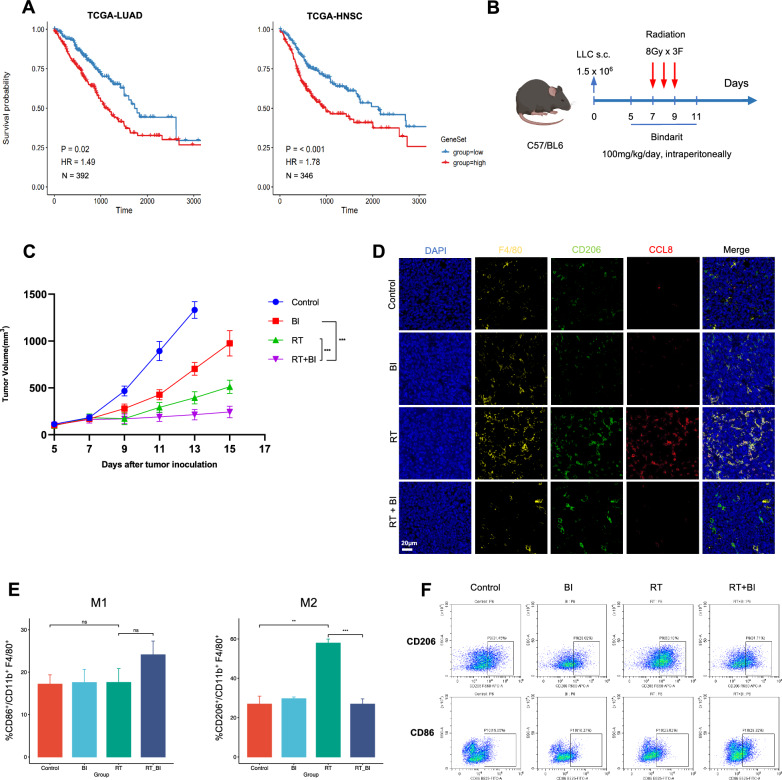
Hypofractionated radiotherapy promotes M2-like Ccl8^high^ macrophages infiltration and leads to poor prognosis. **A** Kaplan–Meier plots showing worse clinical prognosis in the LUAD and HNSC patients with the higher expression level of the Mac_Ccl8 signature. HR, hazard ratio. **B** Schematic diagram of the combination treatment with hypofractionated radiotherapy and the Bindarit. The intraperitoneally administration of Bindarit began from day 5 to day 11 post-tumor injection, and radiation treatment was initiated from day 7 to day 9 post-tumor injection. **C** Growth curves of tumors in LLC-bearing mice in the indicated treatment groups (n = 5 mice/group). Data are presented as mean ± SD with two-way ANOVA test. **D** Representative examples in the indicated treatment groups of multiplex immunofluorescent labeling F4/80, CD206 and CCL8. Yellow, F4/80, Green, CD206; Red, CCL8; Blue, DAPI. **E** Percentages of M1 and M2 macrophages in the specific treatment groups analyzed by flow cytometry (n = 3/group). **F** Representative flow cytometry panels showing M2 macrophages (top) and M1 macrophages (bottom). BI, Bindarit; RT, Radiation therapy; RT + BI, the combination therapy of the radiation and the Bindarit

To investigate whether Mac_Ccl8 in hypofractionated radiotherapy treated s.c. LLC murine model shared similarities with a certain subset in human, we mapped the Ccl8^high^ macrophages signature to well-established myeloid cell atlas – the Pan-myeloid datasets [20]. Surprisingly, the result from the Pan-myeloid dataset supported that the Mac_Ccl8 signature from our dataset was correlated to the human Macro_C1QC subset, and similarly, human Macro_C1QC was indeed classified as M2 state and exhibited phagocytosis function (Additional file 1: Fig. S2D). Thus, we conducted survival analysis by applying expression profile and clinical data from the TCGA-LUAD. The result suggested that high expression of Mac_Ccl8 gene signatures was associated with worse survival outcomes in LUAD and HNSC patients (Fig. 6A).

We further assessed the antitumor effects of the CCL signal (CCL8, CCL7, and CCL2) inhibitor Bindarit in combination with hypofractionated radiotherapy in s.c. LLC murine model. We found that hypofractionated radiotherapy combined with Bindarit significantly prolong the period of local tumor control relative to hypofractionated radiotherapy alone (P < 0.001, Fig. 6B, C). By immunostaining, we confirmed a marked influx of CD206^+^ and CCL8^+^ macrophages following hypofractionated radiotherapy alone relative to combination treatment, with most pronounced infiltration of M2-like Ccl8^high^ macrophages seen after hypofractionated radiotherapy alone (Fig. 6D). We then collected s.c. LLC tumors at early-stage after radiation to evaluated myeloid populations by flow cytometry. The combination therapy reduced the M2 macrophage population and increased the M1 macrophage population as compared to hypofractionated radiotherapy alone (Fig. 6E).

## Discussion

In this study, we successfully identified a distinct M2-like population of macrophage with high expression of Ccl8 level at early-stage post-treatment of hypofractionated radiation in the s.c. LLC murine model, which is considered as immunologically cold tumors. Remarkably, hypofractionated radiation not only promoted CCL8^high^ macrophages infiltration but also contributed to M2-like CCL8^high^ macrophages reprogramming, including upregulated immunosuppressive genes (such as C1qb, Mmp9, and Lgals3bp), downregulated antigen-presenting genes (such as H2-Eb1, H2-Aa, and H2-Ab1), and a crosstalk with T cells via immune checkpoint ligands Lgals9 and Cd86, leading to cytotoxic T cell exhaustion and poor prognosis of the patients. Mechanistically, hypofractionated radiation amplified CCL signaling-dependent cellular communication in the tumor immune microenvironment, thus further enhancing pro-tumorigenic functions of the CCL8^high^ macrophage population. Indeed, hypofractionated radiation in combination with Bindarit, a CCL signal inhibitor, reduced M2-like Ccl8^high^ macrophages infiltration and extended the duration of local tumor control in the LLC-bearing mice. Several findings emerged from our analysis that, if further explored, may help to better understand the underlying mechanisms of converting immunologically cold tumors into hot state, and this novel combination treatment strategy potentially develops new vantage points of NSCLC cancer therapy.

TAMs are implicated in promoting tumorigenesis both at primary and metastatic sites. They facilitate tumor cell growth, invasion, angiogenesis and suppress cytotoxic T cells and natural killer (NK) cells, contributing to immune evasion [12–14]. Consequently, TAM infiltration post-radiotherapy is a critical factor in the establishment of an immunosuppressive environment, leading to radiotherapy resistance. Zhang et al. [5] have comprehensively reviewed the mechanisms underlying TAMs recruitment within radiation. Preclinical studies have shown that radiotherapy promotes CCL2, CSF, and HIF-CXCR4 signaling pathways enhancing TAM infiltration. Our findings are consistent with these reports, demonstrating upregulation of the CSF signaling pathway at early-stage following hypofractionated radiotherapy. Notably, our data suggest a significant role for both CCL8 and CCL2 in TAMs recruitment. Leveraging single-cell RNA sequencing, we identified a specific TAM subpopulation, Mac_Ccl8, which is reprogrammed by hypofractionated radiotherapy. Characterized by high Ccl8 expression, Mac_Ccl8 facilitates the recruitment of other macrophages via the CCL signaling pathway. A recent study has highlighted hypofractionated radiotherapy-induced senescence signatures in macrophages, including genes such as Ccl8, Ccl2, Ccl7, Apoe, and Csf1r [45]. The Mac_Ccl8 subpopulation in our study also highly expressed these genes, which relates with worse overall outcomes in NSCLC patients. Collectively, these insights position Mac_Ccl8 as a potential therapeutic target to mitigate the immunosuppressive impact of hypofractionated radiotherapy in tumor microenvironment.

Chemokines are recognized as crucial cytokines in shaping tumor microenvironment through the recruitment of immune cells such as TAMs, MDSCs, and lymphocytes [12, 14, 46]. Under inflammatory condition, chemokines also induce macrophage polarization towards either M1 or M2 phenotypes [47]. Nevertheless, the mechanisms underlying radiation related immune responses mediated by chemokines remain unclear. Contrary to findings in a glioblastoma research where CCL8 secreted by TAMs was shown to promote tumor growth and invasion [31], our in vitro studies did not demonstrate a similar effect of CCL8 on the growth or invasion of LLC cells. Bindarit is a small molecule inhibitor, which targets CCL signals (CCL8, CCL7, and CCL2) by downregulating the NF-κB pathway [48]. Given that hypofractionated radiotherapy enhanced Ccl8^high^ macrophages infiltration and upregulated CCL signals in a s.c. LLC murine model, we explored a combined therapy of hypofractionated radiotherapy and Bindarit to mitigate TAM infiltration. This combination reduced M2 macrophage infiltration, suggesting that hypofractionated radiotherapy elicits a crosstalk between Ccl8^high^ macrophages and other myeloid cells via the CCL signaling pathway, triggering a cascade effect that recruits and polarizes TAMs. The combination therapy disrupts this cascade effect, thereby prolonging local control of hypofractionated radiotherapy.

Single-cell RNA sequencing offers high resolution transcriptional profiles, yet this study acknowledges three primary limitations. First, while cellular communication analysis indicated an upregulation of the Galectin-9 pathway between Ccl8^high^ macrophages and lymphocytes, the minimal lymphocyte infiltration in LLC tumors constrained our ability to confirm the role of Ccl8^high^ macrophages in T cell exhaustion. Second, the subcutaneous murine tumor model may not fully replicate the tumor microenvironment characteristics observed in orthotopic models. Third, in this study, we only delved on the early alterations in the tumor microenvironment following hypofractionated radiotherapy. Therefore, future studies should address these limitations. For further research directions, the following suggestions could be considered: First, the role of Ccl8^high^ macrophages in other tumor cell lines, particularly those with substantial T cell infiltration, should be validated. Second, it would be informative to assess whether hypofractionated radiotherapy regulates macrophage CCL8 expression through transcription factors. Third, investigating the dynamic changes in the tumor immune microenvironment at different time points following treatment contributes to a better understanding of the immunomodulatory effects of hypofractionated radiotherapy.

## Conclusions

In summary, we have identified a distinct M2-like population of TAMs which marked by highly expressed Ccl8 gene. Hypofractionated radiotherapy reprogrammed Ccl8^high^ macrophages through the upregulation of the CCL signaling pathway which further contributed to TAMs recruitment and polarization. The Ccl8^high^ macrophages infiltration increased at early-stage following hypofractionated radiotherapy and related to treatment resistance. The combination therapy of hypofractionated radiotherapy and CCL signals inhibitor mitigated M2 TAMs infiltration and extended local control. These results highlight that targeting TAMs can synergize with hypofractionated radiotherapy to improve treatment outcomes.

### Supplementary Information

**Additional file 1: Figure S1.** Transcriptional characteristics of LLC cells. **A** UMAP plot showing the representative samples in two groups. **B** UMAP plot showing the distribution of tumor cells and immune cells. **C** Heatmap showing the inferCNV analysis results. **D** Violin plots displaying the gene expression level in the CSF and SPP1 signaling pathways across different clusters. **E** Chord plot and **F** heatmap displaying the CSF signaling pathway network. **Figure S2.** Transcriptional characteristics of myeloid cells. **A** Differentially expressed genes of each myeloid populations. Red dots represent upregulated genes, Blue dots represent downregulated genes. **B** Violin plots showing the gene expression level of the M1 and M2 signatures in the NT and the RT groups. **C** Dot plot showing the KEGG analysis results of macrophages and monocytes. **D** Representative of the Mac_Cc8 signature enrichment in the panmyeloid database (http://panmyeloid.cancer-pku.cn/). **D** Dot plot showing cellular communication signaling pathways between Mac_Ccl8 and other cell types. **F** Hierarchy plot showting the CCL signaling pathway network between myeloid cell populations. **G** Bar plot and **H** heatmap showing the differential interaction number and strength between the NT and the RT groups. **Figure S3.** Cell–cell communication analysis between Mac_Ccl8 and lymphocytes. **A** Dot plot showing the KEGG analysis results of different lymphocytes. **B** Dot plot showing the signaling pathways between Mac_Ccl8 and lymphocytes. **C** Chord plots showing the PD-L1, CD86, and GALECTIN signaling pathway networks. **D** Strength of interactions in the NT and RT groups. **E** Heatmap showing differential communication strength of MHC-I signaling between the NT and the RT groups. **Figure S4.** Recombinant CCL8 protein did not promote LLC cells proliferation in vitro. **A** Cell Viability in the CCK-8 assay in the indicated CCL8 protein concentration group. **B** Colony formation assay for LLC cells with the addition of different concentrations of CCL8 protein concentrations. **C** The gating strategy for isolating macrophages. Live cells were gated first. Then M1 macrophages were gated on CD45^+^CD11b^+^F4/80^+^CD86^+^ population. M2 macrophages were gated on CD45^+^CD11b^+^F4/80^+^CD206^+^ population.

**Additional file 2: Table S1.** Signature genes for defining stemness states and macrophage phenotypes.

## Data Availability

The datasets generated and analysed during the current study are available in the GEO (Gene Expression Omnibus) repository, https://www.ncbi.nlm.nih.gov/geo/query/acc.cgi?acc=GSE256051.

## Abbreviations

TAMs: Tumor-associated macrophages
LLC: Lewis lung carcinoma
NSCLC: Non-small cell lung cancer
SBRT: Stereotactic body radiation therapy
DAMPs: Damage-associated molecular patterns
TANs: Tumor-associated neutrophils
MDSCs: Myeloid-derived suppressor cells
S.C.: Subcutaneous
TME: Tumor microenvironment
RT: Radiation treatment
NT: Radiation naive
IFN-γ: Interferon gamma
GO: Gene Ontology
DEGs: Differentially expressed genes
GSEA: Gene set enrichment analysis
