# Supplementary material for: Single-cell RNA sequencing reveals recruitment of the M2-like CCL8high macrophages in Lewis lung carcinoma-bearing mice following hypofractionated radiotherapy
Source: J Transl Med. 2024 Mar 25;22:306. doi: 10.1186/s12967-024-05118-6 (PMC10964592; doi:10.1186/s12967-024-05118-6)
Supplement: Supplementary file 1 — Additional file 1: Figure S1. Transcriptional characteristics of LLC cells. A UMAP plot showing the representative samples in two groups. B UMAP plot showing the distribution of tumor cells and immune cells. C Heatmap showing the inferCNV analysis results. D Violin plots displaying the gene expression level in the CSF and SPP1 signaling pathways across different clusters. E Chord plot and F heatmap displaying the CSF signaling pathway network. Figure S2. Transcriptional characteristics of myeloid cells. A Differentially expressed genes of each myeloid populations. Red dots represent upregulated genes, Blue dots represent downregulated genes. B Violin plots showing the gene expression level of the M1 and M2 signatures in the NT and the RT groups. C Dot plot showing the KEGG analysis results of macrophages and monocytes. D Representative of the Mac_Cc8 signature enrichment in the panmyeloid database (http://panmyeloid.cancer-pku.cn/). D Dot plot showing cellular communication signaling pathways between Mac_Ccl8 and other cell types. F Hierarchy plot showting the CCL signaling pathway network between myeloid cell populations. G Bar plot and H heatmap showing the differential interaction number and strength between the NT and the RT groups. Figure S3. Cell–cell communication analysis between Mac_Ccl8 and lymphocytes. A Dot plot showing the KEGG analysis results of different lymphocytes. B Dot plot showing the signaling pathways between Mac_Ccl8 and lymphocytes. C Chord plots showing the PD-L1, CD86, and GALECTIN signaling pathway networks. D Strength of interactions in the NT and RT groups. E Heatmap showing differential communication strength of MHC-I signaling between the NT and the RT groups. Figure S4. Recombinant CCL8 protein did not promote LLC cells proliferation in vitro. A Cell Viability in the CCK-8 assay in the indicated CCL8 protein concentration group. B Colony formation assay for LLC cells with the addition of different concentrations of CCL8 protein conce [file 12967_2024_5118_MOESM1_ESM.pdf]

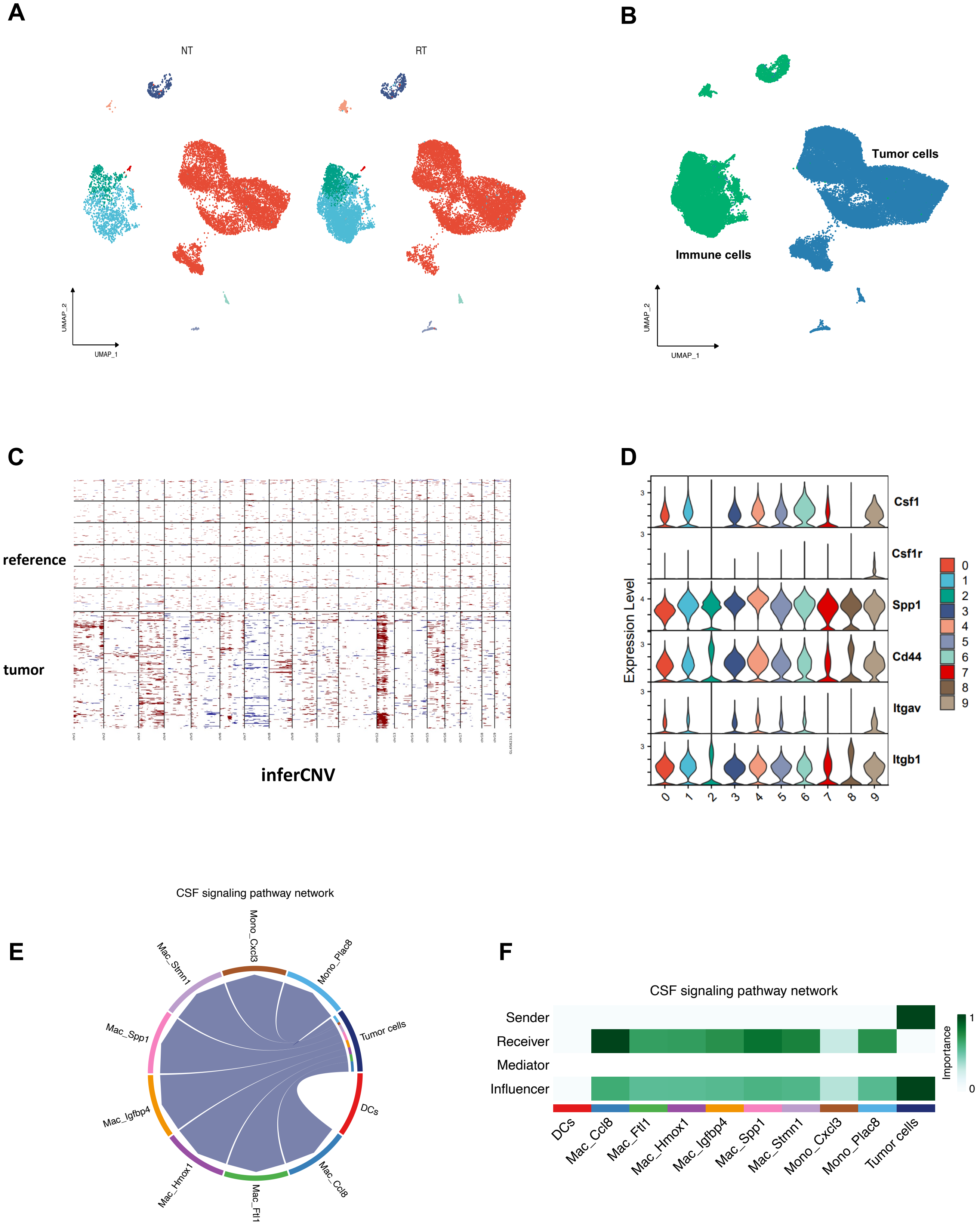
A
NT
RT
UMAP_2
UMAP_1
B
Tumor cells
Immune cells
UMAP_2
UMAP_1
C
reference
tumor
inferCNV
D
Expression Level
Csf1
Csf1r
Spp1
Cd44
Itgav
Itgb1
0
1
2
3
4
5
6
7
8
9
E
CSF signaling pathway network
Mono_Cxcl3
Mono_Plac8
Tumor cells
DCs
Mac_Ccl8
Mac_Ftl1
Mac_Hmox1
Mac_Igfbp4
Mac_Spp1
Mac_Stmn1
F
CSF signaling pathway network
Sender
Receiver
Mediator
Influencer
Importance
1
0
DCs
Mac_Ccl8
Mac_Ftl1
Mac_Hmox1
Mac_Igfbp4
Mac_Spp1
Mac_Stmn1
Mono_Cxcl3
Mono_Plac8
Tumor cells

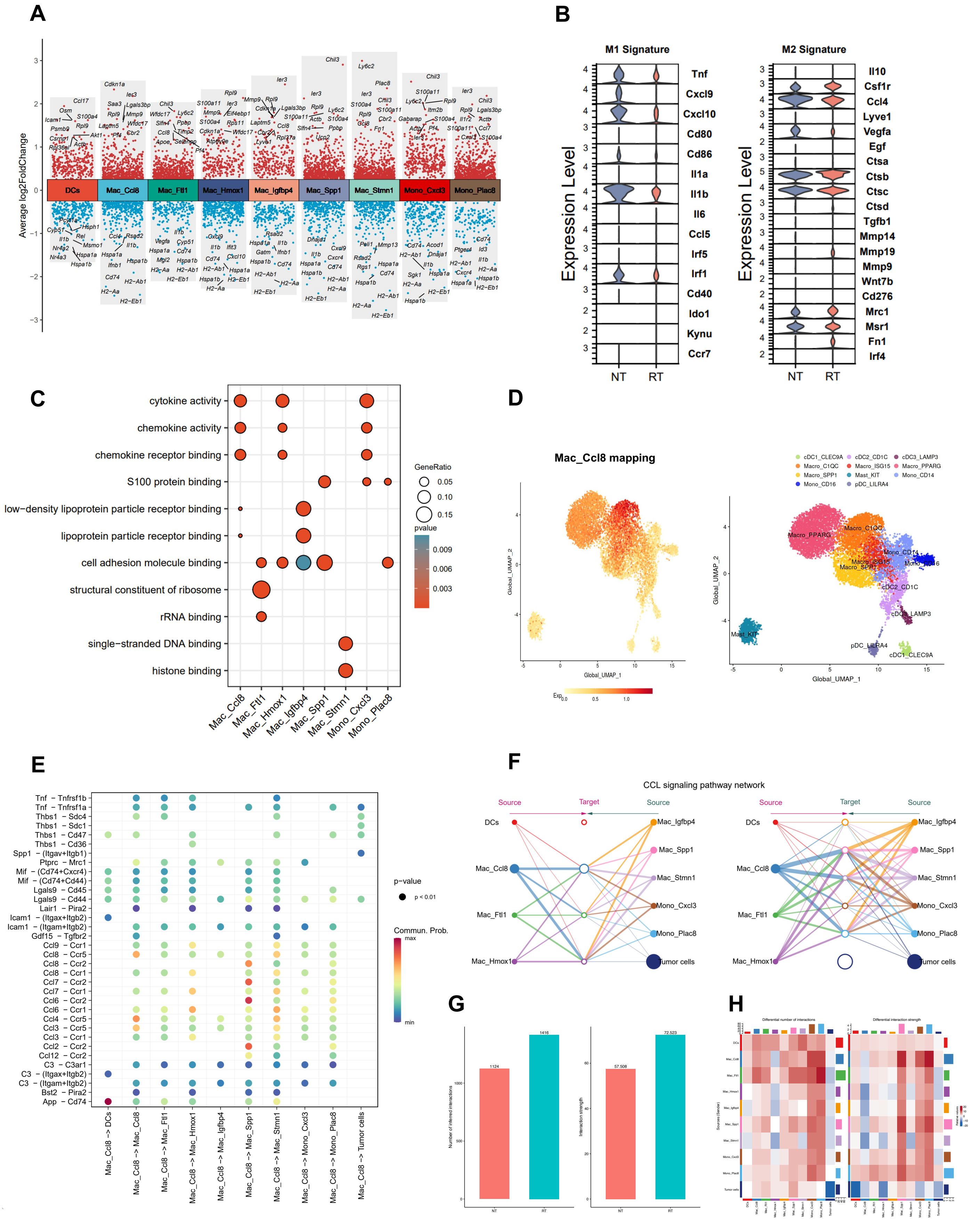

A
Average log2FoldChange
DCs
Mac_Ccl8
Mac_Ftl1
Mac_Hmox1
Mac_Igfbp4
Mac_Spp1
Mac_Stmn1
Mono_Cxcl3
Mono_Plac8
B
M1 Signature
M2 Signature
Expression Level
Tnf
Cxcl9
Cxcl10
Cd80
Cd86
Il1a
Il1b
Il6
Ccl5
Irf5
Irf1
Cd40
Ido1
Kynu
Ccr7
Il10
Csf1r
Ccl4
Lyve1
Vegfa
Egf
Ctsa
Ctsb
Ctsc
Ctsd
Tgfb1
Mmp14
Mmp19
Mmp9
Wnt7b
Cd276
Mrc1
Msr1
Fn1
Irf4
NT
RT
C
cytokine activity
chemokine activity
chemokine receptor binding
S100 protein binding
low−density lipoprotein particle receptor binding
lipoprotein particle receptor binding
cell adhesion molecule binding
structural constituent of ribosome
rRNA binding
single−stranded DNA binding
histone binding
GeneRatio
0.05
0.10
0.15
pvalue
0.009
0.006
0.003
Mac_Ccl8
Mac_Ftl1
Mac_Hmox1
Mac_Igfbp4
Mac_Spp1
Mac_Stmn1
Mono_Cxcl3
Mono_Plac8
D
Mac_Ccl8 mapping
Global_UMAP_1
Global_UMAP_2
cDC1_CLEC9A
cDC2_CD1C
cDC3_LAMP3
Macro_C1QC
Macro_ISG15
Macro_PPARG
Macro_SPP1
Mast_KIT
Mono_CD14
Mono_CD16
pDC_LILRA4
E
p-value
p < 0.01
Commun. Prob.
max
min
Mac_Ccl8 -> DCs
Mac_Ccl8 -> Mac_Ccl8
Mac_Ccl8 -> Mac_Ftl1
Mac_Ccl8 -> Mac_Hmox1
Mac_Ccl8 -> Mac_Igfbp4
Mac_Ccl8 -> Mac_Spp1
Mac_Ccl8 -> Mac_Stmn1
Mac_Ccl8 -> Mono_Cxcl3
Mac_Ccl8 -> Mono_Plac8
Mac_Ccl8 -> Tumor cells
F
CCL signaling pathway network
Source
Target
Source
DCs
Mac_Ccl8
Mac_Ftl1
Mac_Hmox1
Mac_Igfbp4
Mac_Spp1
Mac_Stmn1
Mono_Cxcl3
Mono_Plac8
Tumor cells
G
Number of inferred interactions
Interaction strength
1124
1416
57.508
72.523
NT
RT
H
Differential number of interactions
Differential interaction strength
Sources (Sender)

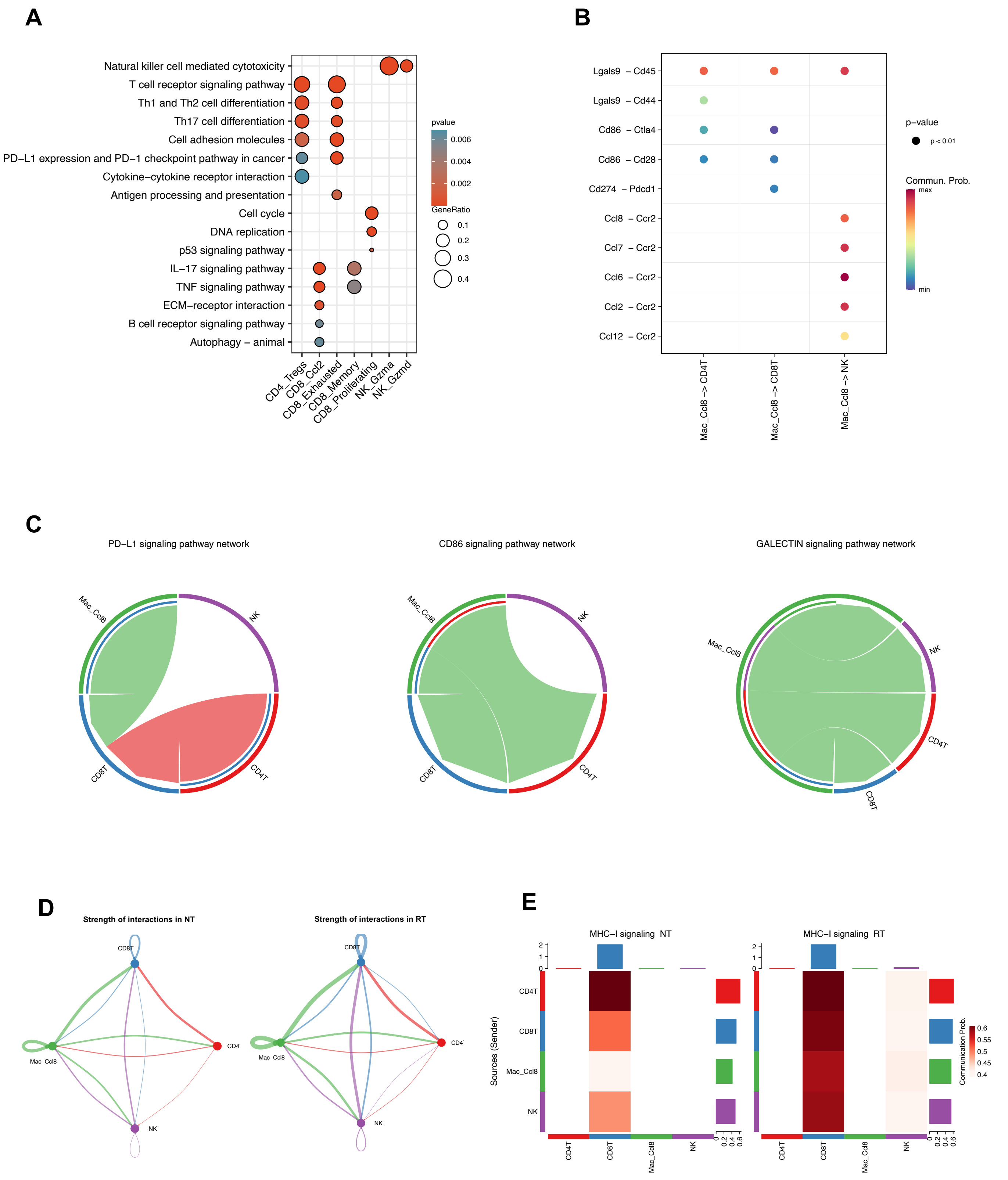
A
Natural killer cell mediated cytotoxicity
T cell receptor signaling pathway
Th1 and Th2 cell differentiation
Th17 cell differentiation
Cell adhesion molecules
PD−L1 expression and PD−1 checkpoint pathway in cancer
Cytokine−cytokine receptor interaction
Antigen processing and presentation
Cell cycle
DNA replication
p53 signaling pathway
IL−17 signaling pathway
TNF signaling pathway
ECM−receptor interaction
B cell receptor signaling pathway
Autophagy − animal
CD4_Tregs
CD8_Ccl2
CD8_Exhausted
CD8_Memory
CD8_Proliferating
NK_Gzma
NK_Gzmd
pvalue
0.006
0.004
0.002
GeneRatio
0.1
0.2
0.3
0.4
B
Lgals9 − Cd45
Lgals9 − Cd44
Cd86 − Ctla4
Cd86 − Cd28
Cd274 − Pdcd1
Ccl8 − Ccr2
Ccl7 − Ccr2
Ccl6 − Ccr2
Ccl2 − Ccr2
Ccl12 − Ccr2
Mac_Ccl8 −> CD4T
Mac_Ccl8 −> CD8T
Mac_Ccl8 −> NK
p−value
p < 0.01
Commun. Prob.
max
min
C
PD−L1 signaling pathway network
CD86 signaling pathway network
GALECTIN signaling pathway network
Mac_Ccl8
NK
CD4T
CD8T
D
Strength of interactions in NT
Strength of interactions in RT
CD8T
CD4T
Mac_Ccl8
NK
E
MHC−I signaling NT
MHC−I signaling RT
Sources (Sender)
CD4T
CD8T
Mac_Ccl8
NK
Communication Prob.
0.6
0.55
0.5
0.45
0.4

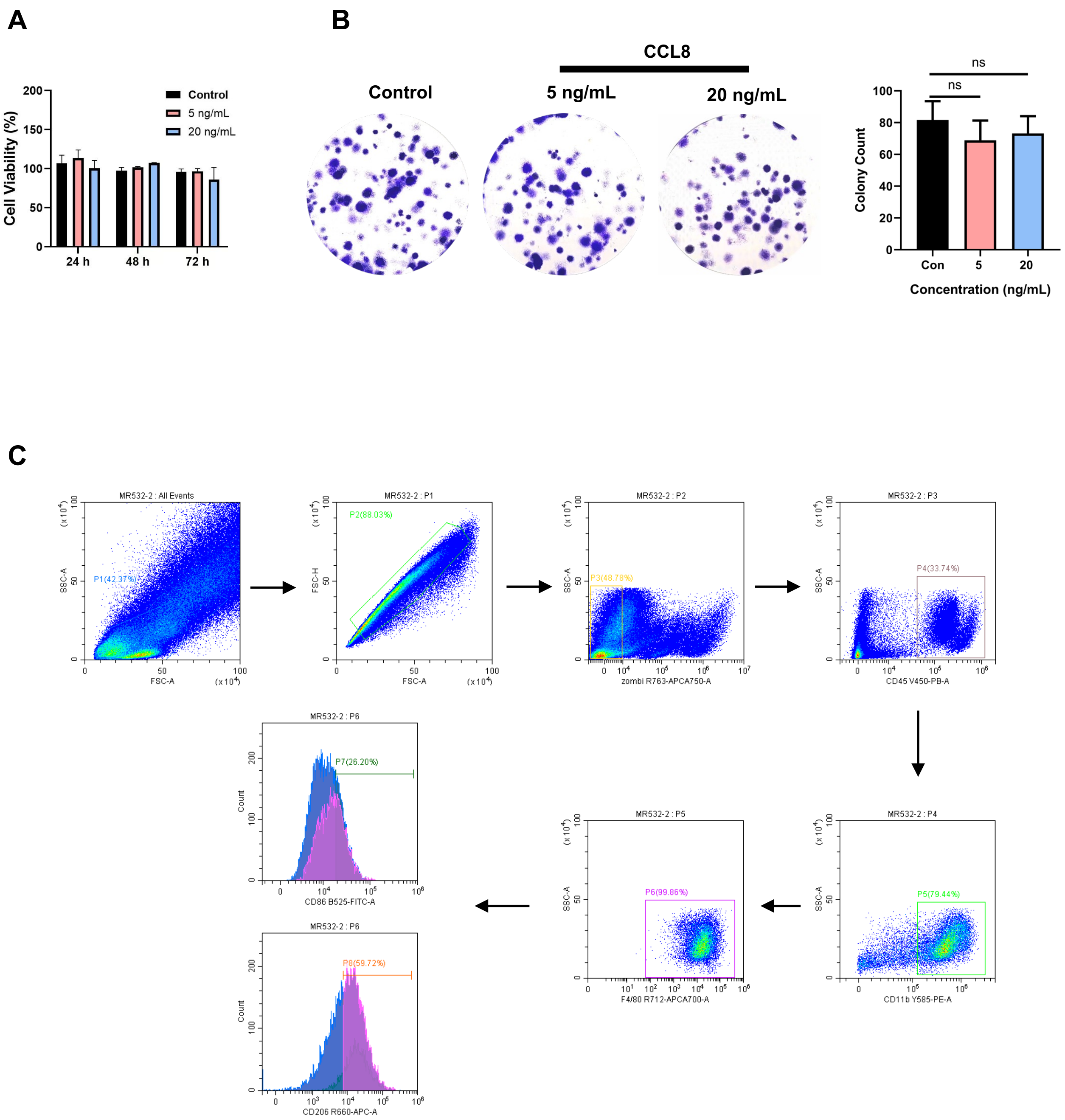

A
Cell Viability (%)
200
150
100
50
0
24 h
48 h
72 h
Control
5 ng/mL
20 ng/mL
B
CCL8
Control
5 ng/mL
20 ng/mL
ns
ns
Colony Count
100
80
60
40
20
0
Con
5
20
Concentration (ng/mL)
C
MR532-2 : All Events
P1(42.37%)
SSC-A
FSC-A
MR532-2 : P1
P2(88.03%)
FSC-H
FSC-A
MR532-2 : P2
P3(48.78%)
zombi R763-APCA750-A
MR532-2 : P3
P4(33.74%)
CD45 V450-PB-A
MR532-2 : P4
P5(79.44%)
CD11b Y585-PE-A
MR532-2 : P5
P6(99.86%)
F4/80 R712-APCA700-A
MR532-2 : P6
P7(26.20%)
Count
CD86 B525-FITC-A
MR532-2 : P6
P8(59.72%)
Count
CD206 R660-APC-A
